# Functional traits variation and adaptive strategies of epilithic bryophytes in degraded karst ecosystem

**DOI:** 10.3389/fpls.2026.1847125

**Published:** 2026-09-07

**Authors:** Xinyue Hu, Yan Lu, Hengbin Zhang, Xiaona Li

**Affiliations:** 1 School of Geography and Environmental Sciences, School of Karst Science, Guizhou Normal University, Guiyang, China; 2 State Engineering Technology Institute for Karst Desertification Control, Guizhou Normal University, Guiyang, China; 3 No. 4 Middle School of Qingzhen, Qingzhen, China

**Keywords:** degraded karst ecosystem, ecological adaptation, epilithic bryophytes, functional traits, interspecific and intraspecific variation

## Abstract

Studies on the variation in plant functional traits provide an important theoretical perspective for elucidating the mechanisms of community assembly and the adaptive strategies of species to their environments. Bryophytes, as pioneer groups undergoing active succession in natural ecosystems, can exert significant ecological benefits, including water retention and soil stabilization, in the ecologically fragile karst regions of southwest China. However, studies on the variation of their functional traits are still insufficient. Therefore, this study selected Guizhou Province (Nidang Stone Forest)-one of the regions most representative of degraded karst ecosystems in Southwest China-as the study area. Focusing on epilithic bryophytes, we aimed to explore the interspecific and intraspecific variation patterns of their functional traits, as well as the adaptive strategies they use to cope with environmental stress through trait trade-offs and combinations. The results showed that the functional traits of epilithic bryophytes exhibit a trait variation pattern of “physiological convergence - morphological differentiation”. Their responses to environmental signals were associated with taking nutrient related traits (C: P ratio, C: N ratio, phosphorus content) as the key hub traits for resource transmission, and the functional integration between morphological traits (increased middle cell vertical wall thickness and middle cell width) and photosynthetic physiological traits (increased non-photochemical quenching, nitrogen content, phosphorus content, calcium content and decreased relative chlorophyll content, maximum photochemical efficiency actual photochemical efficiency of PS II, C: P ratio, C: N ratio, N: P ratio). Such trait variations may enhance nutrient immobilization, potentially contributing to the ecological restoration and sustainable development of degraded karst ecosystems. This study deepens the understanding of functional traits variation in bryophytes, thereby furnishing a theoretical basis for forecasting the adaptive mechanisms of epilithic bryophytes in response to environmental stress and their potential ecological roles across degraded karst ecosystems. It also offers a theoretical reference for the rehabilitation of these ecosystems.

## 1 Introduction

As biological attributes developed over the long-term evolution of plants, plant functional traits reflect the adaptive strategies of plants in key life processes, such as the carbon cycle, water transport, and nutrient cycling (Kumari et al., 2021; Zhang et al., 2020). Trait-based ecological studies have achieved important progress in understanding the functional responses of communities to changes in various environmental factors (Aguirre-Gutiérrez et al., 2019; Kimball et al., 2016). Environmental factors are associated with significant interspecific and intraspecific differences in their impacts on organisms (Jung et al., 2010; Lepš et al., 2011). These effects shape the functional composition and structure of communities by driving changes in species turnover (including relative species abundance and species occurrence frequency) and/or intraspecific variation caused by phenotypic plasticity and genetic differentiation (Wang et al., 2022; Luo et al., 2016). Species turnover is widely recognized as the main driver of community functional structure (McGill et al., 2006). Analyzing the variation patterns of functional traits and their environmental drivers provides a theoretical foundation for clarifying the mechanisms of community assembly and predicting the adaptive responses of species to environmental changes (Blonder et al., 2023). The multi-scale spatial variations in plant functional traits and their environmental drivers, along with the adaptive responses of vegetation communities, have emerged as key research frontiers in ecology in recent years (Moor et al., 2017).

Plant functional traits can reflect a species’ adjustments in growth strategies to environmental changes, either independently or synergistically, while also exerting a profound impact on ecosystem functioning (Cardinale et al., 2012; De Laender et al., 2016). Such covariation is regarded as an effective tool for predicting the effects of environmental changes on plant communities and ecosystem functions (Pan et al., 2023; Cui et al., 2020; Kovenock and Swann, 2018). The recently emerging Plant Trait Networks (PTNs) reveal the linkages among multi-trait synergistic variations, providing a crucial perspective for elucidating the adaptive mechanisms by which plants optimize resource utilization in different habitats (Niinemets, 2015). Constructing a visual correlation network among traits can uncover the intrinsic relationships between individual traits and the overall functional trait system, deepen the understanding of plant survival strategies in dynamic environments, and thereby provide a more solid theoretical foundation for research on ecosystem stability (De La Riva et al., 2016; Kramer-Walter et al., 2016).

With their unique morphological structures and physiological adaptive mechanisms, bryophytes have successfully colonized a wide range of extreme environments, including humidity, drought, high temperature, low light, intense ultraviolet radiation, and severe cold. Some groups even exhibit a truly cosmopolitan distribution pattern (Medina et al., 2011; Schofield and Crum, 1972). Against the background of global climate change and ecosystem degradation, the ecological functions of bryophytes have received increasing attention (Delgado-Baquerizo et al., 2025; Turetsky et al., 2012; Wilsey et al., 2009). On the one hand, as pioneer species in primary succession, bryophytes play a key role in maintaining ecosystem functions by driving carbon and nitrogen biogeochemical cycling, promoting soil formation and nutrient retention, purifying water quality, and regulating microclimate (Liu J et al., 2023; Sáez-Ardura et al., 2025; Meng et al., 2026). On the other hand, due to the absence of vascular tissues, bryophytes rely on direct absorption of water and nutrients through their surface. This feature endows them with rapid and sensitive responses to changes in the chemical composition of air, water, and soil, making them excellent bioindicators for environmental change monitoring (Désamoré et al., 2012; Hylander et al., 2002). More importantly, bryophytes optimize the allocation strategies of key resources such as nutrients and carbon through synergistic variation of morphological and physiological traits. Research on trait covariation provides an important perspective for revealing their adaptive mechanisms in response to environmental gradients at a mechanistic level (Sheng et al., 2023; Waite and Sack, 2010; Grau-Andrés et al., 2022).

As one of the largest contiguous karst regions worldwide, the karst area in Southwest China is characterized by high rock exposure, thin and infertile soils, severe nutrient deficiency, and poor water-holding capacity, with high ecosystem vulnerability and strong habitat heterogeneity (Zhou et al., 2017; Liu et al., 2016; Parise et al., 2009). These conditions are associated with limited plant diversity and strong filtering effects on species composition. Epilithic bryophytes in degraded karst ecosystems can promote carbonate rock weathering and initial soil formation via mechanical disruption by rhizoids and organic acid secretion (Chen et al., 2021; Tu et al., 2021). They can also improve soil fertility by adsorbing nutrients from atmospheric deposition and rock surfaces. Their interception and adsorption functions contribute to soil stabilization and water retention on rocky surfaces, creating favorable conditions for seed germination and growth of other vascular plants, thus serving as a vital component of biodiversity in karst landscapes (Zhang et al., 2023).

Although studies on the functional traits of bryophytes have been increasing in recent years, research on the functional traits of epilithic bryophytes in karst regions remains limited (Coe et al., 2024). Therefore, this study selected the Nidang Stone Forest in Guizhou Province—a region in southwestern China’s karst ecosystem that exemplifies the most severe degradation—as the study area. Taking the epilithic bryophyte species within this region as the research subjects, we systematically measured 31 functional traits, including plant, leaf, and stem traits, as well as photosynthetic physiology and nutrient traits. We aimed to unravel the adaptive strategies of epilithic bryophytes in highly heterogeneous habitats at three levels: First, to analyze the patterns of interspecific and intraspecific variation in functional traits of epilithic bryophytes; second, to reveal the organizational patterns of synergistic variation by constructing trait networks; finally, to identify the driving mechanisms of environmental factors on trait variation, and elucidate the ecological adaptive strategies of epilithic bryophytes under the combined stress of high light intensity, high temperature, low canopy density, and low nutrient availability. Based on this, we propose the following hypotheses: (a) Interspecific variation is the primary source of differences in the functional traits of epilithic mosses in degraded karst habitats; (b) Key environmental factors in degraded karst ecosystem significantly affect trait variation in epilithic bryophytes. Moreover, when facing environmental changes, the functional trait network, particularly its hub traits, plays a dominant role in regulating trait coordination and adaptive responses. The findings will provide a new perspective for exploring the survival strategies of bryophytes in extreme habitats and offer an important theoretical reference for vegetation restoration and ecological management of degraded karst ecosystems.

## 2 Materials and methods

### 2.1 Study sites and bryophyte sampling

**Figure 1:**
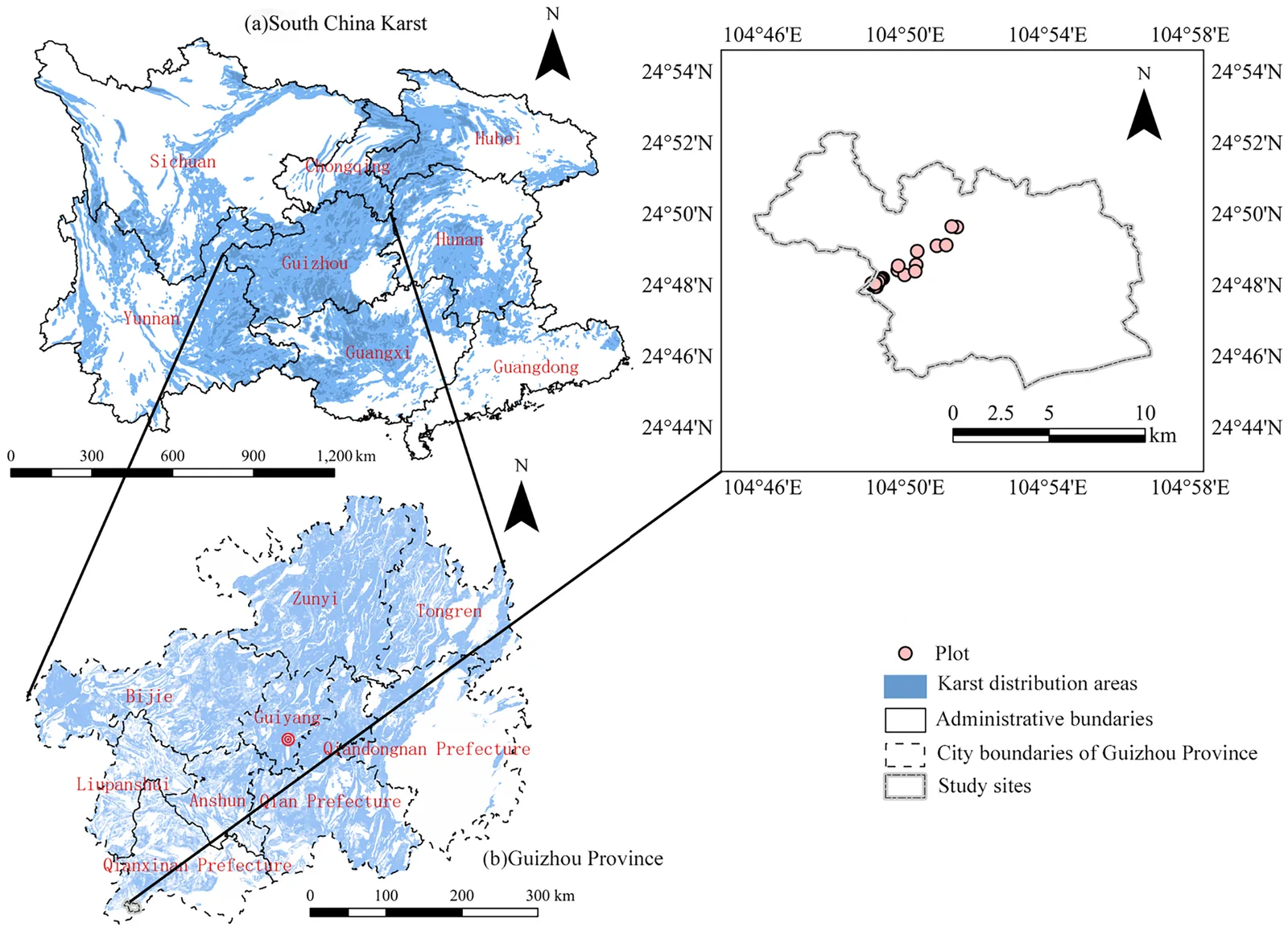
Distribution of epilithic bryophyte sampling sites in Nidang Stone Forest, Guizhou, China.

Guizhou Province is the core distribution area of karst landforms in China. In this study, Nidang Stone Forest, a typical degraded karst region with an area of approximately 8 km², was selected as the study area for sampling (**Figure 1**). Nidang Stone Forest and its surrounding degraded karst areas are located in Nidang Town, southwestern Xingyi City, and belong to the karst peak-cluster depression landform. The elevation ranges from 1100 to 1400 m. The annual average temperature is about 16 °C, with more than 300 sunny days per year and an annual sunshine duration of approximately 3000 h. The annual rainfall is around 1300 mm, and the annual frost-free period lasts 333–344 days. The climate is generally mild and humid, belonging to a subtropical humid monsoon climate.

In August 2022, after excluding sites with intense human disturbance, five 20 m × 20 m plots were established in each of five habitat types in Nidang Stone Forest: non-rocky desertification(NRD), potential rocky desertification(PRD), light rocky desertification (LRD), moderate rocky desertification (MRD), and severe rocky desertification (SRD), yielding a total of 25 plots with sequential numbering (**Supplementary Table 1**). In each plot, three bare rocks representative of the local habitat and community were selected, and two 20 cm × 20 cm epilithic bryophyte subplots were set on the surface of each rock, resulting in a total of 150 subplots. In each subplot, a 10 cm × 10 cm patch of the bryophyte community that exhibited the highest coverage and the healthiest growth status (determined by visual assessment) was collected and stored in an open, transparent bag. After being transported to the laboratory, impurities were removed from the samples, and each sample was divided into four parts: one for the measurement of morphological traits, one for the determination of chlorophyll fluorescence parameters, one for the analysis of plant nutrient element contents, and one for permanent specimen preservation.

The collected bryophyte samples were air-dried in a well-ventilated area and then sorted according to plot type. For species identification, 5–10 shoots were selected and immersed in clean water for 3–5 minutes until the leaves were fully expanded. Temporary slides were prepared and examined under a microscope for morphological observation. Morphological characteristics of the shoots, leaves, cells, and leaf margins were observed using an Olympus SZX-16 stereomicroscope (Olympus Corporation, Japan) and an Olympus BX53 light microscope (Olympus Corporation, Japan), together with standard dissecting tools. Species identification was performed following classical morphological taxonomic methods (Chinese Academy of Sciences, Editorial Committee of Flora Bryophytorum Sinicorum, 2017). All specimens were identified to the species level. For specimens that were difficult to identify, photographs were taken and the identification was confirmed through consultation with relevant experts (**Supplementary Table 2**).

### 2.2 Environmental factors measurements

In this study, the microenvironmental factors at the growth sites of all epilithic bryophyte samples were measured in the field. A GPS device was used to record the geographic coordinates and elevation of the center of each plot. Meanwhile, a compass was used to determine the slope position (SP) of each plot, and a clinometer was used to measure the slope gradient (Slope), slope aspect (Aspect), and rock inclination angle. During the sampling campaign in August 2022, on clear, cloud-free afternoons (12:00–15:00), the instruments were placed 1–2 cm above the bryophyte surface to measure air temperature (Ta) and air humidity (Ha) using a handheld thermohydrometer (MDM25, Michell, UK); light intensity (Light) was measured using a portable illuminance meter (MAVOLUX 5032, Gossen, Germany); and UV radiation was measured using a UV-A radiometer (Photoelectric Instrument Factory of Beijing Normal University, China). Rock surface temperature (RSTa) was measured by pressing an infrared thermometer (DL333600, Deli, China) directly against the rock surface. To reduce random error, each parameter was measured three times per subplot, and the mean of the three measurements was used as the final value for that sampling day. The lithology of the growth substrate was identified using the dilute hydrochloric acid test, and the substrate was confirmed to be carbonate rock. A complete list of all environmental factors with their codes and descriptions is provided in **Table 1**.

**Table 1:** Morphological traits (shoot, leaf, stem, and cell levels), physiological traits (nutrient content and chlorophyll fluorescence parameters) and environmental factors measured for epilithic bryophytes.

Variables	Code	Description
Shoot level	SH	Height of a single bryophyte shoot, determined in cm
Leaf level	LL	Axial length from the leaf tip to the leaf base of a single leaf, measured in μm
	LW	Maximum width per leaf, μm
	L L/W	Ratio of leaf length to width for a single leaf
	LA	Total surface area of a single leaf, assessed in μm²
Stem level	SER	Ratio of the stem epidermal layer width to the total stem diameter of a single shoot
	SCR	Ratio of the stem cortical layer width to the overall stem diameter of an individual shoot
Cell level	MCL	Length of cells in the middle region of a single leaf, measured in μm
	MCW	Width of cells in the middle of a single leaf, measured in μm
	MC L/W	Aspect ratio of cells located in the mid-leaf region of an individual leaf
	MC_V_WT	Longitudinal wall thickness of mid-leaf cells
	MC_H_WT	Transverse wall thickness of mid-leaf cells
	BCL	Length of cells in the base of a single leaf, measured in μm
	BCW	Width of cells in the base of a single leaf, measured in μm
	BC L/W	Aspect ratio of cells situated in the leaf base region of an individual leaf
	BC_V_WT	Longitudinal wall thickness of basal leaf cells
	BC_H_WT	Transverse wall thickness of basal leaf cells
Nutrient content	C	Total carbon accumulation of the bryophyte shoot, in g/kg
	N	Total nitrogen accumulation of the bryophyte shoot, in g/kg
	P	Total phosphorus accumulation of the bryophyte shoot, in g/kg
	K	Total potassium content of the bryophyte shoot, in g/kg
	Ca	Total calcium accumulation of the bryophyte shoot, in g/kg
	Mg	Total magnesium accumulation of the bryophyte shoot, in g/kg
	C: N	Proportion of total carbon content to total nitrogen content in the bryophyte shoot
	C:P	Proportion of total carbon content to total phosphorus content in the bryophyte shoot
	N:P	Proportion of total nitrogen content to total phosphorus content in the bryophyte shoot
Chlorophyll fluorescence parameters	SPAD	The relative chlorophyll content
	Fv/Fm	Quantum yield of PS II photochemistry in the dark-adapted state (equivalent to [Fm Fo]/Fm)
	qP	Assessing the efficiency of photochemical energy conversion in plant photosynthesis
	NPQ	Mechanisms by which plants dissipate excess excitation energy through non-photochemical pathways under high light conditions
	ФPS II	Quantum yield of PS II photochemistry in the light-adapted state (equivalent to [Fm’ − Fs]/Fm’)
Environmental factors	Elevation	Altitude of the sampling subplot, measured in meters (m)
	TRASP	Aspect transformed to a linear scale from 0 (south‑facing) to 1 (north‑facing)
	CD	Percentage of canopy cover directly above the sampling point (%)
	Ta	Air temperature 5 cm above the rock surface, measured in °C
	Ha	Relative air humidity 5 cm above the rock surface, measured in %
	RSTa	Temperature of the bare rock surface adjacent to the bryophyte patch, measured by infrared thermometer (°C)
	Light	Photosynthetically active radiation (or illuminance) at the rock surface, measured in lux (or μmol·m^–2^·s^–1^)

Codes for each variable are listed in the table for ease of reference in subsequent descriptions.

### 2.3 Morphological and physiological traits measurements

For each bryophyte sample, five mature and healthy individuals were selected for morphological trait measurements at the shoot, leaf, stem, and cell levels (**Table 1**). The samples were first rinsed with ultrapure water and then rehydrated at room temperature for 30 min until fully expanded. Shoot height (SH) was measured as the natural extended length from the shoot base to the apex using a digital calliper (Mitutoyo 500‑196‑30, Mitutoyo Corporation, Japan; precision: 0.01 mm). Three healthy and intact leaves were detached from the upper one‑third portion of each individual. Leaf images were captured using an Olympus BX53 microscope (Olympus Corporation, Tokyo, Japan) equipped with an Olympus DP74 digital camera (Olympus Corporation, Tokyo, Japan). For stem anatomical measurements, freehand cross‑sections were prepared from the middle segment of the main stem. To calibrate the image scale, a stage micrometer (1 mm divided into 100 divisions, 0.01 mm per division) was placed on the microscope stage, and an image of the micrometer scale was captured under the same magnification and camera settings used for sample images. In the ImageView software, a line was drawn along a known distance on the micrometer image to establish the pixel‑to‑distance ratio, thereby completing the scale calibration. Image analysis was then performed to measure morphological trait values and calculate the proportions of related tissues. All morphological data are presented as the mean of five replicate measurements.

Physiological traits measured in this study mainly included nutrient contents and chlorophyll fluorescence parameters (**Table 1**). For nutrient content determination, fresh bryophyte samples were subjected to enzyme inactivation at 105 °C for 30 min, and then dried in a 75 °C thermostatic oven to constant weight. After cooling, the dried bryophyte samples were fully ground into powder in an agate mortar. After homogenization, 50 mg of the powder was accurately weighed, packed into plastic sealed bags, and stored for subsequent analysis of nutrient element indicators. Among these, C content was determined using a German Vario-MAX elemental analyzer; N content was determined using the Kjeldahl method. For potassium (K), calcium (Ca), magnesium (Mg), and phosphorus (P), dried samples (0.2 g, ground to <0.15 mm) were digested in a microwave digestion system (CEM MARS 6, Matthews, NC, USA) with 5 mL concentrated HNO_3_ (65%, GR) and 2 mL H_2_O_2_ (30%). The digestion program was: ramp to 120 °C in 5 min and hold for 5 min; then ramp to 180 °C in 5 min and hold for 15 min. After cooling, the digest was diluted to 25 mL with ultrapure water (resistivity ≥18.2 MΩ·cm). Concentrations of K, Ca, Mg, and P were measured using an Inductively Coupled Plasma Optical Emission Spectrometer (ICP-OES, Thermo iCAP 7000 Series, Thermo Fisher Scientific, Waltham, MA, USA) at wavelengths of 766.490 nm (K), 317.933 nm (Ca), 279.553 nm (Mg), and 213.618 nm (P). Calibration curves were prepared from multi‑element standard solutions (0, 1, 5, 10, 20, 50 mg/L for K, Ca, Mg; 0, 0.5, 1, 2, 5, 10 mg/L for P).

We selected healthy individuals with intact moss crowns and fully expanded, naturally green leaves to conduct *in situ* SPAD measurements at the field sampling sites. All measurements were conducted on clear, cloud‑free afternoons (12:00–15:00). A chlorophyll meter (SPAD-502Plus, Konica Minolta, Japan) was used to measure the upper middle part of the moss crown. For each sample, ten individuals were measured, each with three replicate readings, and the mean of all measurements was used as the final SPAD value for that sample. Chlorophyll fluorescence parameters were determined in the laboratory. Individual bryophyte samples were rehydrated in darkness for 24 hours and then subjected to 30 min of dark adaptation. Initially, the samples were exposed to a weak measurement light (< 0.1 μmol·m^–2^·s^–1^), and the initial fluorescence (F_0_) of a single bryophyte leaf was measured using a basic-model modulated chlorophyll fluorometer (JUNIOR-PAM, Shanghai Zequan Technology Co., Ltd.). Next, a saturation pulse light (10000 μmol·m^–2^·s^–1^) was applied for 0.8 s to determine the maximum fluorescence under dark adaptation (Fm). Subsequently, the samples were fully light-adapted for 30 min at an actinic light intensity of 250 μmol·m^–2^·s^–1^. The steady-state fluorescence (Fs) was recorded, and another saturation pulse was applied to measure the light-adapted maximum fluorescence (Fm’). The light was then turned off, and far-red light was immediately switched on for 5 s to determine the light-adapted minimum fluorescence (F_0_’). Based on the above measurements, the following parameters were calculated: variable fluorescence (Fv), maximum photochemical efficiency of PSII (Fv/Fm), photochemical quenching (qP), non-photochemical quenching (NPQ), and actual photochemical efficiency of PS II (ФPS II). For each sample, 10 individuals were measured with 3 replicates per individual, and the mean value was used. The detailed calculation formulas are shown in Equations 1–4:

(1) $$Fv/Fm=(Fm-F_{0})/Fm$$

(2) qP=(Fm'−Fs)/(Fm'−F0')

(3) NPQ=Fm/Fm'−1

(4) ФPSII=(Fm'−Fs)/Fm'

### 2.4 Statistical analysis

For a given trait, interspecific trait variation was quantified by calculating the Coefficient of Variation (CV) of mean trait values across species. For a given species and trait, intraspecific trait variation was quantified by calculating the CV of mean trait values of the species population across all sampling plots. The CV was calculated as (Standard Deviation/Mean). To further quantify the relative contributions of interspecific versus intraspecific variation to total trait variance, we performed variance partitioning using linear mixed models (LMM) with species as a random effect (Albert et al., 2010). Specifically, the model was specified as Trait ~ 1 + (1 | Species), where the global intercept (1) represents the overall trait mean, and the random intercept for each species ((1 | Species)) allows species-specific deviations from this mean; the variance of these deviations directly quantifies interspecific variation. All variance components were estimated using the Restricted Maximum Likelihood (REML). The proportion of total variance explained by interspecific differences and within-species variation was calculated for each trait.

To explore the organizational patterns of synergistic variation in epilithic bryophytes, a correlation network was constructed based on the screened 31 functional traits. All statistical analyses and network construction were performed using R software 4.5.0. To ensure the reliability of network correlations and the clarity of network structure, we set *P* < 0.05 and |*r*| > 0.4 as screening thresholds. This threshold was selected because trait coordination in bryophytes is relatively loose and susceptible to environmental fluctuations; applying a more lenient threshold (|*r*| > 0.2) would produce an overly dense network with indistinct modules, whereas |*r*| > 0.4 yields a clearer modular structure. On this basis, an undirected weighted network of functional traits in epilithic bryophytes was constructed based on the Pearson adjacency matrix. Visualization of the network structure was achieved using the Fruchterman-Reingold force-directed layout algorithm. In the network, nodes represented single functional traits, and the weight of edges between nodes directly reflected the magnitude of Spearman’s rank correlation coefficient, intuitively indicating the strength of trait correlations. Network characteristic analyses, including computational programming and result output, were completed using the igraph package in R. Edge Density (ED) and Modularity index (Q) were selected to characterize the overall connectivity properties of the network. Community detection was performed using the fast-greedy algorithm implemented in the cluster-fast-greedy function of the igraph package. To evaluate the statistical robustness of the detected modules, we performed 1000 case-dropping bootstrap resampling iterations, the modular structure was stable in over 95% of the iterations (**Supplementary Table 3**). Three indicators, degree centrality (directly reflecting the number of connections of a single trait node), betweenness centrality (the number of shortest paths passing through the node, betweenness), and eigenvector centrality (quantifying the core influence of a trait node in the entire network), were used to quantify the network centrality of each functional trait (Flores-Moreno et al., 2019). To verify the structural stability of the final trait correlation network, a bootstrap resampling test with 1000 random sample eliminations was conducted in this study to ensure the reliability and repeatability of the network topological results (**Supplementary Table 4**).

Furthermore, to determine the relationships between the functional traits of epilithic bryophytes and environmental driving factors, the correlation coefficients between variables were calculated using the corr correlation analysis function in R 4.5.0. Meanwhile, a correlation plot was generated using the corrplot visualization package (method = “spearman”) to intuitively visualize the trait-environment factor associations. The correlation plot clearly reflected the strength and positive/negative direction of associations among various variables, and efficiently identified the key environmental driving factors with significant effects on the variation of bryophytes’ functional traits (α = 0.05). To explore and visualize the linear relationships between functional traits and the core environmental driver, we used the prcomp and lm functions in R 4.5.0 and extracted regression statistics with the broom package. Specifically, we first performed principal component analysis (PCA) on the seven environmental factors, then constructed univariate linear regression models using the first principal component (PC1) as the independent variable and each of the 31 functional traits as the dependent variable. All data were standardized prior to analysis to eliminate the effects of different units of measurement.

## 3 Results

### 3.1 Variation in the functional traits of epilithic bryophytes

The patterns of variation in the functional traits of bryophytes showed significant differentiation between and within species, with the coefficient of variation ranging from 5% to 92% between species and from 4% to 37% within species (**Table 2**). Except for SPAD, qP, P, and Ca content, the coefficient of variation between species was higher than the coefficient of variation within species for all other traits. At the interspecific level, BC L/W (Basal Cell Length/Width) had the highest coefficient of variation (92%), while NPQ had the lowest (5%). In addition, MC L/W (Middle Cell Length/Width) (77%), SH (Shoot Height)(72%), MCL (Middle Cell Lengh) (67%), L L/W (Leaf Length/Width) (54%), and LA (Leaf Area) (52%) all showed high levels of interspecific variation (**Table 2**). At the intraspecific level, P and C:P exhibited the highest coefficient of variation, reaching 37%, while Fv/Fm and NPQ had the lowest intraspecific coefficients of variation, both at 4% (**Table 2**). The variance partitioning results further revealed that for the vast majority of morphological traits (MCL, MC L/W, L L/W, SH, LL, MCW (Middle Cell Lengh), BCL (Basal Cell Length), BCW (Basal Cell Width), BC L/W (Basal Cell Length/Width)), the proportion of interspecific variance reached 65–92%, whereas for MCvWT (Longitudinal wall thickness of mid-leaf cells), BCvWT (Longitudinal wall thickness of basal leaf cells), and BC_H_WT (Transverse wall thickness of basal leaf cells), the interspecific proportion was only 7–20%. Among photosynthetic physiological traits, the intraspecific variance proportion exceeded 94% for all photosynthetic parameters (SPAD, Fv/Fm, NPQ, ΦPSII, and qP). For most nutrient traits (P, Ca, C:P, N:P), the intraspecific proportion was as high as 84 – 97%, with interspecific variation being negligible.

**Table 2:** Interspecific and intraspecific variation coefficients (CV, %) and variance proportion (%) of functional traits for epilithic bryophytes(values are presented as interspecific/intraspecific).

Morphological traits		CV/%	Variance proportion (%)	Physiological traits		CV/%	Variance proportion (%)
Shoot	SH	72/30	77/23	Chlorophyll fluorescence parameter	SPAD	12/14	3/97
Leaf	LL	34/14	76/24		Fv/Fm	6/4	3/97
	LW	33/22	63/37		NPQ	5/4	6/94
	LA	52/28	47/53		qP	17/19	0/100
	L L/W	54/16	91/9		ФPSII	7/6	4/96
Stem	SER	24/19	41/59	Nutrient content	N	19/18	21/79
	SCR	9/6	46/54		C	14/12	59/41
Cell	MCVWT	20/18	18/82		P	37/37	6/94
	MCHWT	21/20	36/64		k	40/27	52/48
	MCL	67/17	92/8		Mg	36/27	37/63
	MCW	33/10	85/15		Ca	20/23	5/95
	MC L/W	77/18	92/8		C:P	44/37	16/84
	BCVWT	25/17	7/93		C:N	21/18	23/77
	BCHWT	32/23	20/80		N:P	30/27	15/85
	BCL	48/21	74/26				
	BCW	31/15	65/35				
	BC L/W	92/28	70/30				

Stating that interspecific variation was calculated based on all species occurring across all plots (150 samples in total), while intraspecific variation was calculated based on the average number of individuals measured per species (ranging from 2 to 26 individuals per species).

### 3.2 Correlation patterns of functional traits in epilithic bryophytes

**Figure 2:**
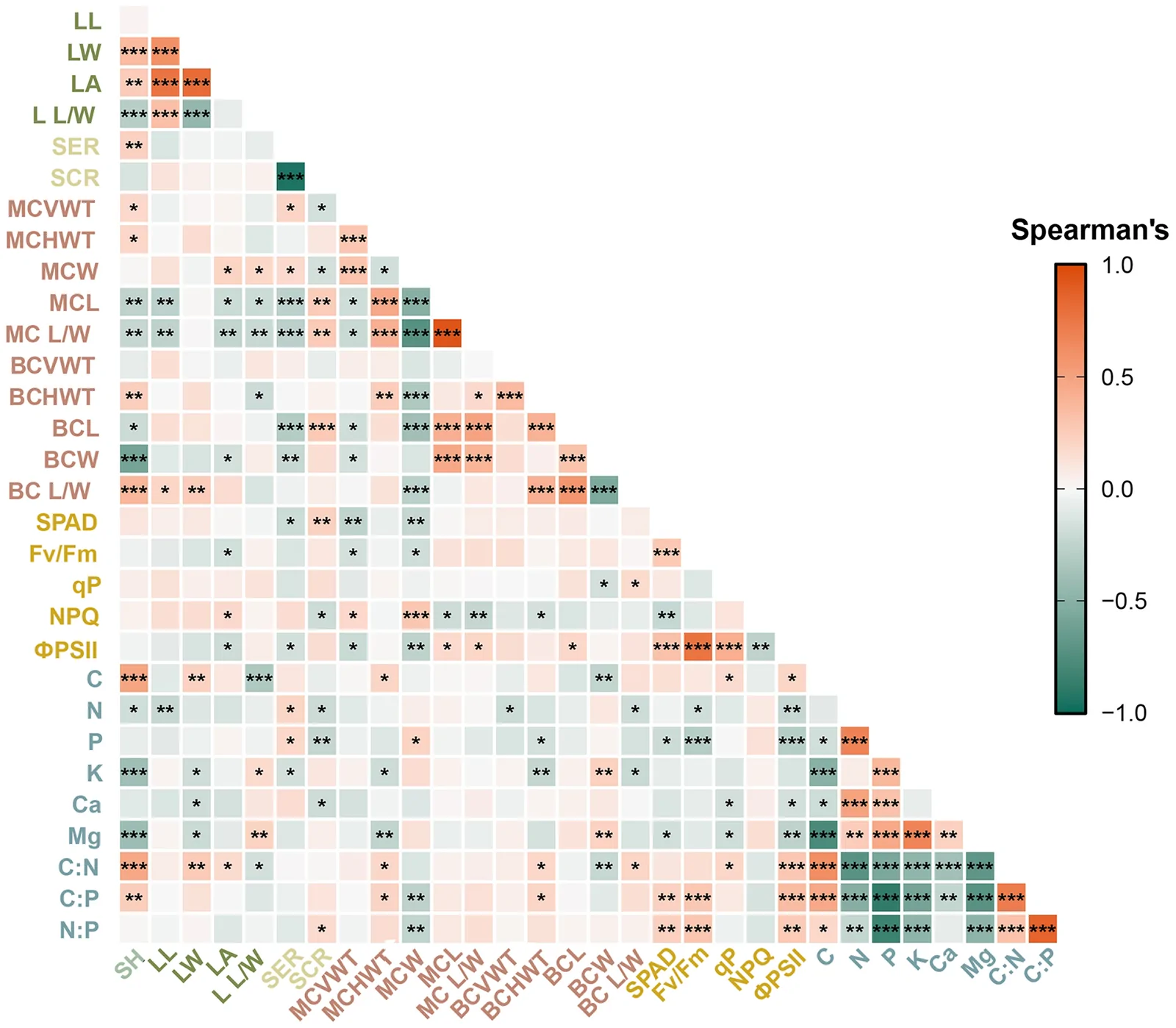
Correlogram depicting the degree of correlation between all measured traits of epilithic bryophytes in the degraded karst ecosystem, including morphological traits, nutrient traits, and chlorophyll fluorescence parameters. Significance levels are marked as: *P < 0.05; **P < 0.01; and ***P < 0.001.

Extensive and significant correlations were detected among functional traits of bryophytes. Specifically, at the shoot level, SH was positively correlated with leaf-level traits (LW (Leaf Width), LA (Leaf Area); *r* = 0.36, 0.25, respectively), SER (Stem Epidermal Ratio) (*r* = 0.22), cellular structural traits (MC_V_WT, MC_H_WT, BC_H_WT, BC L/W; r = 0.18 to 0.38), and nutrient traits (C, C:N and C:P; r = 0.24−0.48), while it was negatively correlated with L L/W (*r* = -0.29), several cell morphological traits (MCL, MC L/W, BCL, BCW; *r* = -0.19−-0.59), and nutrient traits (N, K and Mg; *r* = -0.17−-0.41) (*P* < 0.05, **Figure 2**). At the leaf level, LA was positively correlated with MCW (*r* = 0.21), NPQ (*r* = 0.18), and C:N (*r* = 0.17), while showing negative correlations with MC L/W (*r* = -0.24), BCW (*r* = -0.16), Fv/Fm (*r* = -0.16), and ФPS II (*r* = -0.19) (*P* < 0.05, **Figure 2**). At the stem level, SER was positively correlated with MC_V_WT (*r* = 0.20), MCW (*r* = 0.17), N (*r* = 0.20), and P (*r* = 0.18), while showing negative correlations with SCR (Stem Cortical Ratio) (*r* = -0.94), several cellular traits (MCL, MC L/W, BCL, and BCW; *r* = -0.22−-0.31), SPAD (*r* = -0.20), ФPS II (*r* = -0.16), and K (*r* = -0.16) (*P* < 0.05, **Figure 2**). At the cell level, MC L/W was positively correlated with BC L/W, BC_H_WT (*r* = 0.17−0.49) and ФPS II (*r* = 0.20), while negatively correlated with NPQ (*r* = -0.23); BC L/W was positively correlated with C: N (*r* = 0.20) and qP (*r* = 0.17), but negatively correlated with N (*r* = -0.19) and K (*r* = -0.21) (*P* < 0.05, **Figure 2**).

### 3.3 Functional trait network of epilithic bryophytes in heterogeneous habitats

**Figure 3:**
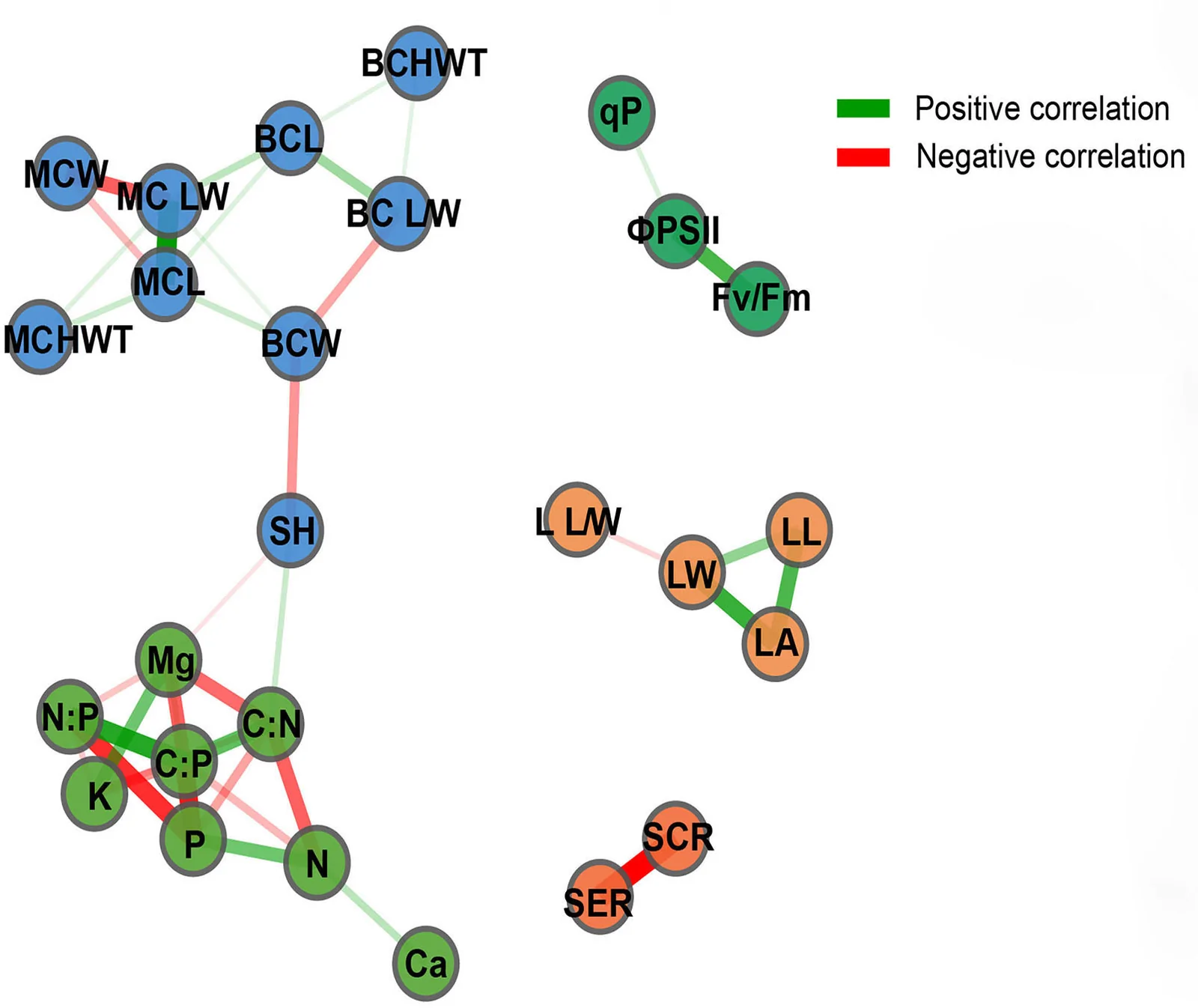
Trait networks of epilithic bryophytes in degraded karst ecosystem. Links between the nodes express significant positive (green) or negative (red) trait correlations, with varying width and saturation proportional to the correlation intensity. The width of the border of a node is proportional to its strength centrality.

The functional trait network of bryophytes comprised 26 nodes and 45 edges (**Figure 3**). Among the modules, the largest module consisted of 9 traits, covering most cellular traits and plant height (SH). The second largest module, comprising nutrient traits, was primarily associated with SH and the largest cluster (**Figure 3**). Network centrality analysis showed that C:P had the highest centrality (Eigen = 1, **Table 3**) and the highest network connectivity (Degree = 6, **Table 3**); C:N, Mg, and P also exhibited centrality (Eigen > 0.79) and relatively high network connectivity (Degree = 5–6) (**Table 3**). In terms of betweenness centrality, BCW had the highest value (Betweenness = 66, **Table 3**), followed by SH (Betweenness = 64, **Table 3**); C:N, Mg, and LL (Leaf Length) also exhibited relatively high betweenness centrality (Betweenness = 26–28, **Table 3**). In addition, MC_V_WT, BC_V_WT, SPAD and NPQ showed no connections in the network (**Table 3**).

**Table 3:** Node parameters of epilithic bryophyte functional trait networks in degraded karst ecosystem.

Trait	Network node parameter			Trait	Network node parameter		
	Degree	Betweenness	Eigen		Degree	Betweenness	Eigen
C:P	6	4	1.000	BCL	4	6	0.006
P	5	2	0.850	MCW	2	0	0.005
C: N	6	28	0.803	MC_H_WT	2	0	0.004
Mg	6	27	0.798	BC_H_WT	2	0	0.002
N:P	4	0	0.710	LL	2	0	0.000
K	4	1	0.587	LW	3	2	0.000
N	4	15	0.544	LA	2	0	0.000
SH	3	64	0.232	L L/W	1	0	0.000
Ca	1	0	0.087	SER	1	0	0.000
BCW	4	66	0.049	SCR	1	0	0.000
MCL	5	26	0.013	Fv/Fm	1	0	0.000
MC L/W	5	11	0.013	qP	1	0	0.000
BC L/W	3	10	0.010	ΦPS II	2	1	0.000

### 3.4 Environmental drivers of functional trait variation in epilithic bryophytes across heterogeneous habitats

**Figure 4:**
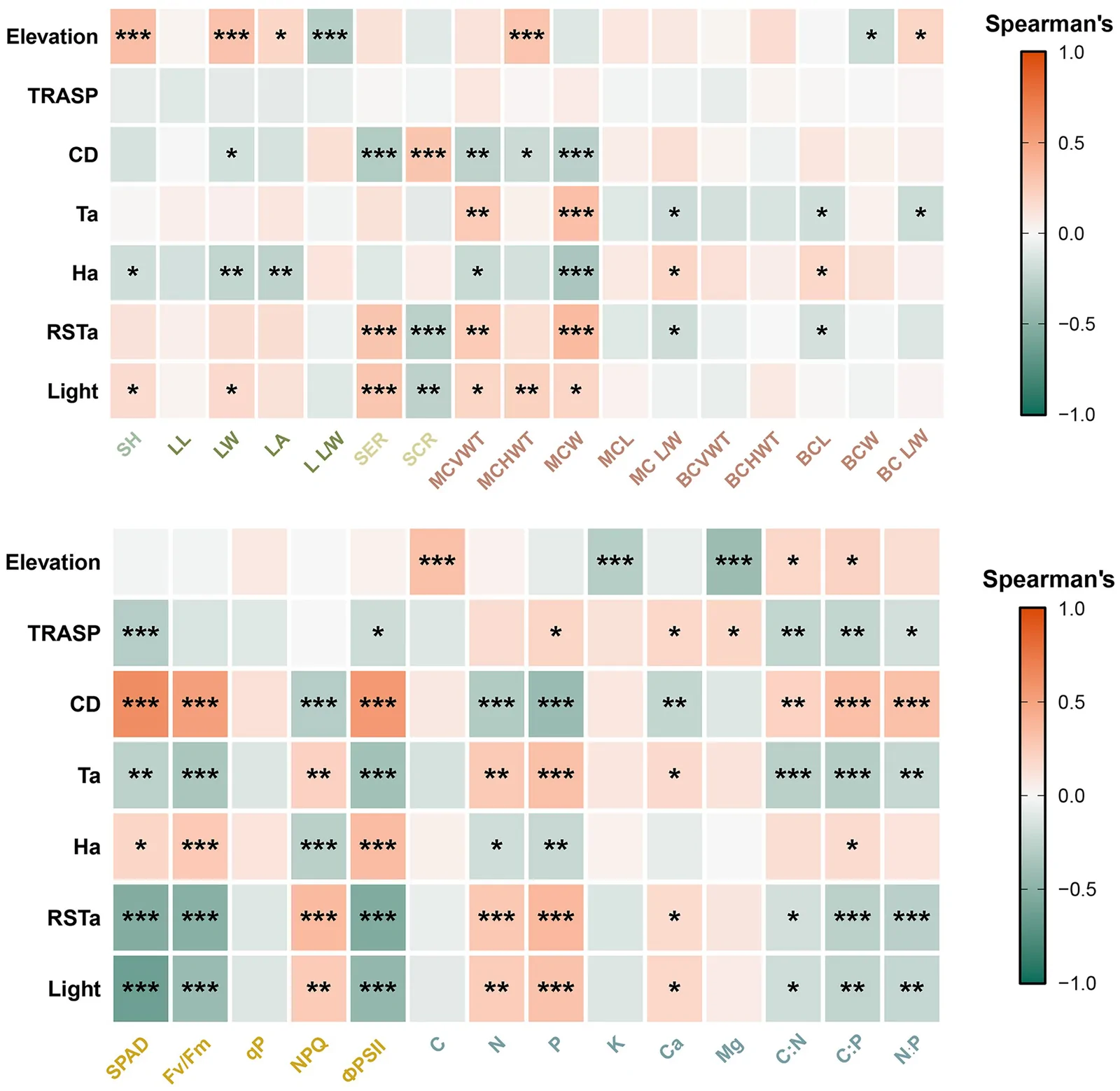
Correlogram plots illustrating the correlation levels among all measured traits of epilithic bryophytes and environmental factors. Significance: *P < 0.05; **P < 0.01; and ***P < 0.001.

Significant correlations were identified between functional traits of bryophytes and various environmental factors. Specifically, Elevation, CD (Canopy Density), Ta (Atmospheric Temperature), RSTa (Rock Surface Temperature), and Light were the primary drivers shaping trait variation in this study (*P* < 0.05, **Figure 4**). Elevation was positively correlated with morphological traits (SH, LW, LA, MC_H_WT, and BC L/W; *r* = 0.20–0.34) and physiological traits (C, C:N, and C:P; *r* = 0.19−0.32), while showing negative correlations with L L/W (*r* = -0.29), BCW (*r* = -0.20), K (*r* = -0.29), and Mg (*r* = −0.41) (*P* < 0.05, **Figure 4**). Morphological traits (MC_V_WT, MCW) and physiological traits (NPQ, N, P, Ca) were positively correlated with Ta (*r* = 0.17−0.33), RSTa (*r* = 0.17−0.37) and Light (*r* = 0.19–0.31), while showing negative correlations with CD (*r* = -0.23−-0.42) (*P* < 0.05, **Figure 4**). Physiological traits (SPAD, Fv/Fm, ФPS II, C:N, C:P, N:P) were positively correlated with CD (*r* = 0.22−0.62) and negatively correlated with Ta (*r* = -0.23−-0.38), RSTa (*r* = -0.16−-0.54), and Light (*r* = -0.18−-0.62) (*P* < 0.05, **Figure 4**).

**Figure 5:**
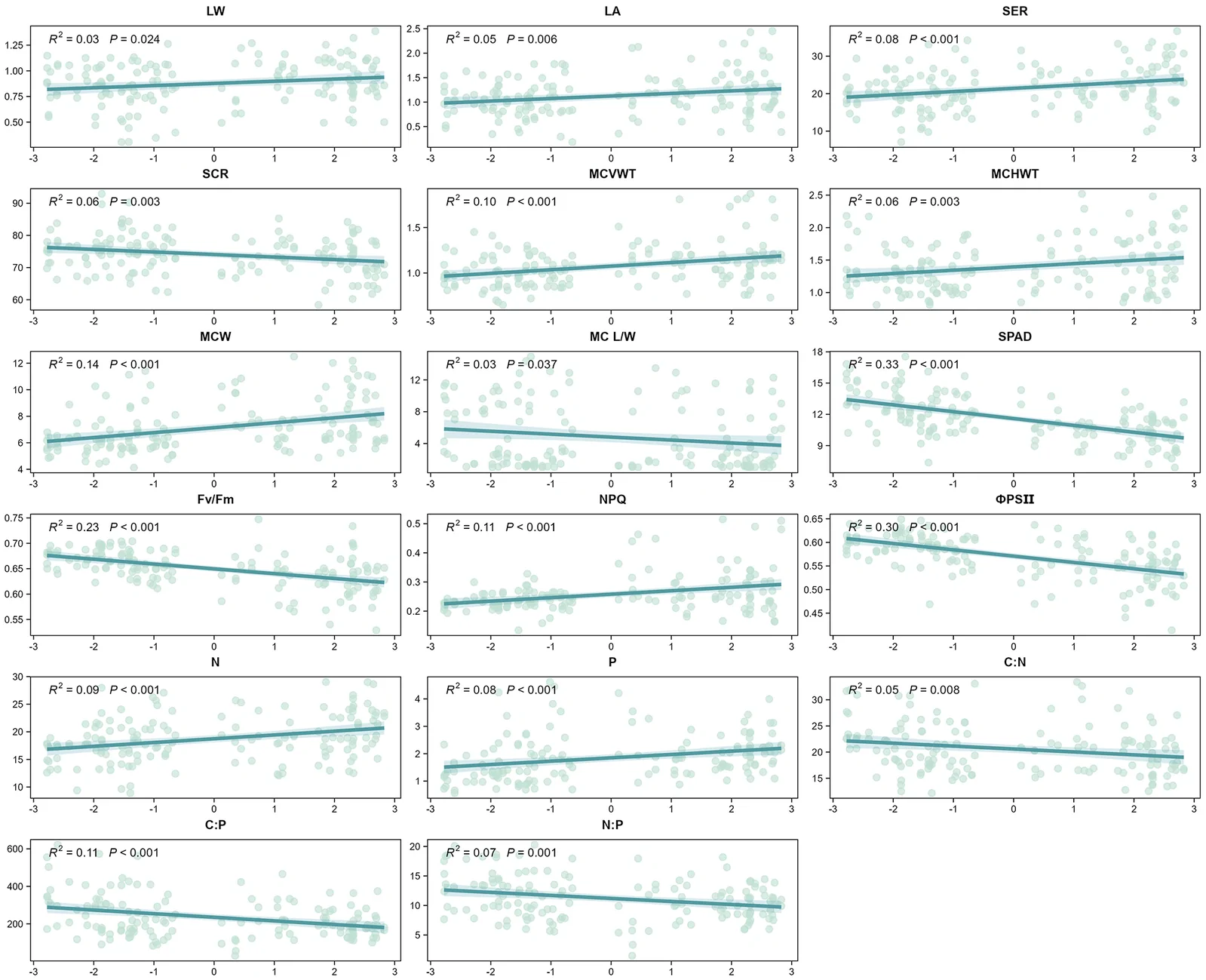
Linear regression analysis revealed the relationships between functional traits of epilithic bryophytes and the synthetic environmental gradient (the first principal component, PC1). PC1 was derived from principal component analysis of seven environmental factors: elevation, topographic solar radiation index (TRASP), canopy density, air temperature, air humidity, rock surface temperature, and light intensity. Higher PC1 values represent stronger degradation stress. Each panel shows a statistically significant linear relationship between a single trait and PC1 (p < 0.05).

PC1 explained 50.24% of the total environmental variance (**Supplementary Table 6**). The loadings of the seven environmental variables on PC1 (**Supplementary Table 7**) indicated that higher PC1 values were primarily associated with lower canopy density (CD: -0.496) and higher rock surface temperature (RSTa: 0.488), light intensity (Light: 0.461), and air temperature (Ta: 0.378). Elevation (0.076) and TRASP (0.111) contributed only weakly. Linear regression analysis revealed that most morphological, photosynthetic, and nutrient traits of epilithic bryophytes were significantly influenced by the environmental gradient PC1 (*P* < 0.05, **Figure 5**). In terms of morphological traits, LW, MCW, and SCR significantly increased with increasing PC1 (*P* < 0.05). Regarding photosynthetic traits, NPQ significantly increased, whereas SPAD, Fv/Fm, and ΦPSII significantly decreased along PC1(*P* < 0.05). For nutrient traits, C:P and N:P ratios significantly increased, while P content significantly decreased with increasing PC1 (*P* < 0.05).

## 4 Discussion

### 4.1 Relative contributions of interspecific and intraspecific variation to functional traits

Functional trait-based community assembly mechanisms generally regard community assembly as a deterministic process (Kraft et al., 2015; Chai et al., 2019), with environmental filtering and ecological niche differentiation as key associated factors (Cornwell et al., 2006). Specifically, environmental filtering screens for species with similar adaptive traits, thereby driving trait convergence among species within the community; under resource-limited conditions, interspecific competition further promotes divergent differentiation of functional traits to achieve niche separation (Jung et al., 2010). Meanwhile, genetic diversity and phenotypic plasticity are associated with divergent trait variation within the same species across a highly heterogeneous degraded karst ecosystem. Thus, a key question remains: which of these two sources of variation—interspecific or intraspecific—dominates the functional traits of epilithic bryophyte communities in degraded karst habitats?

The variance partitioning results (**Table 2**) show that for most cellular traits (MCL, MCW, MC L/W, BC L/W), as well as leaf length/width ratio (L L/W) and shoot height (SH), the interspecific variance proportion reached 76–92%. In contrast, for all chlorophyll fluorescence parameters (SPAD, Fv/Fm, NPQ, ΦPSII) and most nutrient traits, the intraspecific variance proportion was as high as 84–100%. This indicates that community-level differences in morphological and most cellular traits are predominantly driven by interspecific variation, whereas intraspecific variation explains a substantial portion of physiological trait variation (**Table 2**). This finding does not fully align with our first hypothesis, which proposed that interspecific variation is the primary source of differences in the functional traits. Interspecific variation has long been widely accepted as the core driver of changes in community functional structure (McGill et al., 2006). However, the results of this study confirm that intraspecific trait variation, driven by phenotypic plasticity and local adaptation, can also significantly shape community functional composition (Violle et al., 2012; Zheng et al., 2024). These findings are consistent with studies along elevational gradients (Roos et al., 2019) and across vertical stratification of tropical montane cloud forest canopies (Tucker et al., 2025), where species turnover dominates some traits while plasticity dominates others. This work further advances our understanding of the mechanisms underlying functional trait variation in bryophyte communities, highlighting that neither interspecific nor intraspecific variation should be overlooked in functional trait studies.

### 4.2 Morphological differentiation and physiological convergence in degraded karst habitats

The expression of plant functional traits is jointly shaped by environmental filtering and inherent genetic background (Tomato Genome Consortium, 2012; Huang et al., 2013). Although species can generate trait variation through phenotypic plasticity and genetic diversity, the magnitude of variation is constrained by their inherent genetic background and plasticity potential, making it far narrower than the broad range of interspecific trait variation afforded by species turnover. Environmental filtering screens and retains species with similar functional traits that are adapted to ambient environmental conditions (Givnish, 1987; De Bello et al., 2009); competition among species subsequently drives niche differentiation, leading to significant divergent evolution of functional traits at the interspecific level. The leaves of the vast majority of bryophytes consist of a single layer of cells (Wright et al., 2017; Carriquí et al., 2019).As the primary functional units of bryophytes, leaf cell structure directly influences water balance and photosynthetic efficiency (Cutler et al., 1977; Drake et al., 2012). This study observed that the leaf cellular traits of epilithic bryophytes (BC L/W, MCL/W, MCL) exhibited significant interspecific variation (coefficient of variation ranging from 67% to 92%), with interspecific variation accounting for as much as 70% to 92% of the total variation, reflecting differences in water acquisition and retention strategies among species. Meanwhile, morphological traits (SH, LA, L L/W) also exhibited high interspecific variation (coefficient of variation ranging from 52% to 72%). Among them, shoot height (SH) and leaf area (LA) directly affect light interception and carbon acquisition capacity (Westoby et al., 2002; Lienin and Kleyer, 2012). These morphological differences constitute diverse strategies of bryophytes in space occupancy and light resource utilization. In the complex and heterogeneous degraded karst ecosystem, epilithic bryophyte species with different genetic backgrounds have diverged in resource use strategies via differentiation in morphology and cellular structure, thereby reducing interspecific competition and enhancing the overall adaptability and coexistence of the community.

Traits related to plant photosynthesis (Fv/Fm, NPQ, ФPS II) showed low interspecific and intraspecific variation (**Table 2**), indicating that photosynthetic function remained highly consistent across different epilithic bryophyte species. In degraded karst ecosystems with simple vegetation community structure and low canopy density, exposed rocks enhance the reflection and scattering of surface sunlight. As a result, all bryophyte species must maintain high photosynthetic capacity to ensure the normal operation of their photosynthetic apparatus, which may be a key reason for the limited variation in their photosynthetic traits. In addition to these convergent responses at the photosynthetic level, we also observed substantial intraspecific plasticity in nutrient-related traits—phosphorus acquisition—which may serve as a complementary mechanism for coping with resource limitation. Within the degraded karst ecosystem characterized by consistently low phosphorus availability (He et al., 2004; Huang et al., 2021; Su et al., 2023), we found that the phosphorus-related nutritional trait (C:P) exhibited the highest intraspecific variation (37%), and its intraspecific variation accounted for 84% of the total variation. This suggests that epilithic bryophyte individuals may adjust their phosphorus uptake strategies via phenotypic plasticity to cope with resource competition and sustain survival, thereby enhancing their local adaptive capacity.

In summary, in karst habitats with limited resources and high spatial heterogeneity, environmental stress drives physiological convergence and significant intraspecific plasticity in bryophytes to ensure basic survival, while at the morphological and structural levels, significant interspecific differentiation and limited intraspecific variation give rise to diverse resource utilization strategies. Such a trait variation pattern of “physiological convergence and morphological differentiation” collectively maintains species coexistence and ecosystem functioning in bryophyte communities. This result indicates that the first hypothesis—that interspecific variation is the primary source of differences in functional structure—holds true at the morphological and cell levels but deviates importantly at the physiological level.

### 4.3 Inter-trait correlation and functional integration of epilithic bryophytes

Plants’ adaptation to environmental changes does not depend on a single trait, but rather on the synergistic interaction of multiple traits. which form a distinct network through tight inter-correlations (Poorter et al., 2013; Kleyer et al., 2019) and jointly underpin the whole-plant survival strategy (Li et al., 2022). In this study, our analysis of the trait network of bryophytes in the Nidang Stone Forest identified five functional modules (**Figure 3**). Significant correlations were detected among these traits (**Figure 2**). The high modularity of the trait network enables plants to adapt more flexibly to complex and variable environments (Alon, 2003). The tight internal connections within each trait module, together with weak associations between different modules, indicate that these modules can act as relatively independent evolutionary units. Synergistic optimization of traits within modules enables epilithic bryophyte communities to cope with diverse environmental stresses in degraded karst ecosystem through functional division of labor, thereby maintaining their overall adaptability (Messier et al., 2010). Such structural features reflect marked functional specialization and intrinsic trade-offs in the adaptive strategies of epilithic bryophytes, providing important evidence to support the second hypothesis proposed in this study.

Our trait network analysis indicated that most leaf cellular traits constituted the largest functional module (**Figure 3**). In bryophytes lacking a vascular system, leaf cellular traits are central to determining water relations and mechanical performance (Proctor, 2000). Variation in leaf cell size can regulate light transmittance and light energy conversion efficiency by affecting the distribution and density of chloroplasts, while also participating in water transport and stress‑tolerance physiological processes (Coe et al., 2019). Close coordination and variation among leaf cellular traits enable specific trade-offs between hydraulic safety and efficiency, making this one of the key mechanisms by which bryophytes adapt to drought stress (Li et al., 1992). Among these traits, basal cell width (BCW) exhibited the highest betweenness centrality (Betweenness = 66, **Table 3**), and was significantly correlated with shoot height (SH), leaf morphological traits (LA), cellular traits (MCvWT, MCL, MC L/W, BC L/W), Stem epidermal ratio (SER), photosynthesis‑related traits (qP), and nutrient traits (C, Mg, K, C: N) (*P* < 0.05, **Figure 2**). Traits with high betweenness centrality are typically located on the connecting pathways between different modules in the trait network. By coordinating and integrating relationships among multiple traits, they exert substantial influences on the overall structure and functioning of the network (He et al., 2020). Notably, although bryophytes lack true vascular tissues, the leaf basal cells are anatomically positioned at the junction between the stem and the leaf blade, where water and nutrients are transferred from the stem to the leaf. The thickening of cell walls may facilitate apoplastic water and solute exchange. Moreover, the leaf base may coordinate local water uptake, mechanical support, and carbon allocation, thereby indirectly influencing the overall resource status of the plant (Song et al., 2024). Based on the unique topological centrality of leaf basal cells in the trait network and their anatomical properties, we hypothesize that bryophytes achieve coordination across multiple functional modules through leaf cell traits, and we propose this as a testable hypothesis for future physiological studies.

Shoot height (SH), which also possessed high betweenness centrality (Betweenness = 64, **Table 3**), connected the largest module composed of the majority of cellular traits and the second-largest module constituted by nutrient traits (**Figure 3**). Shoot height (SH) was significantly correlated with leaf morphological traits (LW, LA, L L/W), stem anatomical traits (SER), leaf cellular traits (MCvWT, MC_H_WT, MCL, MC L/W, BC_H_WT, BCL, BCW, BC L/W), and nutrient traits (C, N, K, Mg, C:N, C:P) (*P* < 0.05, **Figure 2**). N and P play a crucial role in metabolic processes such as protein and nucleic acid synthesis and energy transfer (Chun et al., 2017). When nutrient levels are low, plants are constrained at the molecular, cellular, tissue, and shoot levels, leading to growth limitations. The increase in shoot height may be restricted by the acquisition and allocation of N, P, and other nutrients, and is also accompanied by higher water transport resistance and mechanical risks, which need to be compensated for by specific cellular structures (Proctor, 2000). Furthermore, plant height exhibits considerable intraspecific variation (72%), further supporting its ecological significance as a key trait for adaptive differentiation among species (He et al., 2025).

A network degree can be used to quantify the importance of a trait within the trait network (Flores-Moreno et al., 2019). Traits with high network degrees play a crucial role in resource transfer and functional integration (Armbruster et al., 2014). Through trait network analysis, this study found that nutrient traits such as C:P, C:N, and P exhibited high network degree and centrality (**Figure 3**). This indicates that these traits are hub traits for bryophytes adapting to the degraded karst ecosystem, and they play a crucial ecological regulatory function. Most previous studies have heavily focused on the environmental adaptability of the macroscopic morphological traits of bryophytes. However, in degraded karst ecosystem characterized by soil nutrient scarcity and instability, the core selective pressure faced by epilithic bryophytes may lie in how to efficiently acquire, allocate, and conserve these extremely limited nutrient resources. For bryophytes, which lack a vascular system and depend strongly on internal nutrient cycling, nutrient traits may perform more fundamental physiological regulatory functions than macroscopic morphological traits under severely nutrient-limited conditions, acting as key chemical hubs linking environmental stress, individual physiology, and ecosystem nutrient cycling (Mohanasundaram and Pandey, 2022). As hub traits, C:P and C:N ratios were significantly correlated with traits at multiple levels, including leaf morphology, cell morphology, and photosynthetic physiology. This suggests that these central hub traits can act as a bridge for functional relationships across different morphological and tissue levels -such as the plant, leaves, and stems and photosynthetic physiology, thereby enabling trait coordination through the integration of morphological and physiological information (Forsum et al., 2008). In addition, the C:P exhibited the highest intraspecific coefficient of variation (37%, **Table 2**), indicating that epilithic bryophytes in P‑limited degraded karst ecosystem possess flexible plasticity in P utilization to adapt rapidly to subtle changes in environmental P availability. This high plasticity provides bryophytes with rapid environmental responsiveness. Combined with its high connectivity and regulatory role in the trait network, it enables bryophytes to efficiently allocate limited resources at the shoot level under stressful conditions, and endows them with ecological adaptability to flexibly integrate environmental signals and coordinate multiple physiological processes (Liu X et al., 2023). These results reflect the evolutionary advantage of nutrient traits as a “decision‑making hub” for adaptation to degraded rocky karst habitats.

### 4.4 Environmental drivers of traits synergy and adaptive strategies in epilithic bryophytes

Plant functional traits serve as a bridge between plants and their environment (Violle et al., 2007), reflect the long-term adaptation of plants to environmental conditions (Grime, 2006), and also exert significant impacts on ecosystem functioning. Variation in functional traits is associated with the regulation of multiple environmental factors (Rao and Terry, 1995). In degraded karst ecosystem, complex and diverse topographic structures result in significant spatial differentiation of environmental factors such as light. In this study, principal component analysis (PCA) was performed on seven environmental factors (elevation, topographic aspect index, canopy density, air temperature, humidity, rock surface temperature, and light intensity), and the first principal component (PC1) was extracted as a synthetic environmental gradient (**Supplementary Tables 6**, **S7**). PC1 was primarily defined by canopy density (negative loading), rock surface temperature, light intensity, and air temperature (positive loadings). Thus, PC1 primarily represents a gradient of increasing thermal and radiation stress associated with decreasing vegetation cover. Linear regression analysis revealed that most morphological, photosynthetic, and nutrient traits were significantly linearly related to PC1 (*P* < 0.05), with photosynthetic traits (SPAD, Fv/Fm, NPQ, ΦPSII) showing particularly strong responses(**Figure 5**). Furthermore, correlation analysis between epilithic bryophyte functional traits and environmental factors further supported this trend, indicating that temperature (Ta, RSTa), canopy density (CD), and light intensity (Light) are key environmental drivers shaping bryophyte trait variation (**Figure 4**, **Supplementary Table 7**). This may be because vegetation degradation represents the most direct indicator and manifestation in degraded habitats (Zhang et al., 2023). Changes in canopy density are usually accompanied by variations in light intensity, temperature, and water evaporation, which in turn affect the living environment of bryophytes. Low canopy density is associated with higher light intensity, drastic rock temperature fluctuations, and more frequent drought stress (Márialigeti et al., 2009). This also explains why photosynthetic traits (SPAD, Fv/Fm, NPQ, ФPS II) are most directly related to resource capture and utilization exhibited the strongest responses to the environment (*P* < 0.05, **Figures 4**, **5**).

With increasing degradation severity, high light intensity, high temperature(Ta and RSTa), low canopy density, and low nutrient availability collectively form a combined stress environment. The synergistic variation in morphological traits of epilithic bryophytes was characterized by increases in SER and cellular traits (MC_V_WT, MCW) and a decrease in SCR (**Figure 1**). Unlike vascular plants, bryophytes lack internal structures for water regulation, and their tissue water content is thus more susceptible to environmental fluctuations (Richardson, 1981). Therefore, by adjusting their cell morphological structure, bryophytes are able to achieve an effective balance between water retention and light interception (Tao and Zhang, 2012; Wu et al., 2001), with morphological alterations in cells also playing a crucial role in carbon exchange processes (Coe et al., 2019). Thickened cell walls (MC_V_WT) may contribute to reducing cellular water loss and enhancing mechanical stability, and are also considered the main sites of light scattering within leaves, which is consistent with the “detour effect” (i.e., the light path between cells is prolonged and homogenized) (Neumann, 1995; Terashima and Saeki, 1983). We propose that thickened cell walls can enhance light scattering at the cell wall–air interfaces, thereby acting as a physical barrier before light reaches the photosynthetic apparatus. This reduces the influx of excess photons to the photosynthetic reaction centers and alleviates high-light stress. Through these cellular-level adaptations, bryophytes are able to improve water use efficiency under stressful conditions and enhance water retention while regulating photosynthesis (Rice and Giles, 1996), thereby indirectly influencing water cycling processes in degraded karst ecosystems and contributing to local ecological restoration. Notably, the observed decrease in SCR and increase in SER with rising light intensity in this study contradict the conclusion of previous research that increasing light promotes a higher cortical ratio to facilitate substance transport (Wu, 1998). This may be explained by epilithic bryophytes reducing the cortical ratio to alleviate damage from excess light energy, while increasing the epidermal ratio to strengthen mechanical stability.

When faced with environmental changes, plants typically adapt their physiological traits to cope with resource constraints (Freschet et al., 2018). The covariation in the photosynthetic traits of bryophytes is characterized by an increase in NPQ and decreases in SPAD, Fv/Fm, and ΦPS II (**Figures 4**, **5**). In habitats with low canopy density, high light, and high temperature, the synergistic variation of bryophyte morphological traits is associated with reduced incident light intensity, likely through physical blocking and light scattering. When light stress is inferred to exceed the structural defense threshold, excess light energy may reach the photosynthetic system. Decreases in ФPS II and Fv/Fm indicate that the activity of PS II reaction centers is inhibited in epilithic bryophytes under such stress, the conversion of light energy captured by photosynthetic pigments into chemical energy is impeded, and light energy conversion efficiency is reduced (Xia et al., 2023). At this point, as an indicator for the dissipation of excess excitation energy (Ruban, 2016), the elevated NPQ suggests that when the open proportion of PS II reaction centers and the energy involved in photochemical reactions are suppressed, excess light energy is dissipated in the form of heat to protect the photosynthetic apparatus (Zhang et al., 2023; Fan et al., 2020). Meanwhile, stress disrupts the structure and function of bryophyte chloroplasts and induces the accumulation of large amounts of free chlorophyll in cells. To mitigate photo-oxidative damage, cells must accelerate the degradation of this chlorophyll, resulting in a significant decrease in SPAD values (Li et al., 2020). These photosynthetic adjustments minimize photodamage at the expense of suppressing individual‑level carbon fixation. Nevertheless, this physiological suppression does not compromise the ecosystem‑scale role of bryophytes as carbon cycle drivers. Despite transient declines in photosynthetic rates per unit biomass during stress, the large standing biomass, extended longevity, and slow turnover of bryophyte communities ensure their continued function as net carbon sinks over annual to decadal timescales. The short‑term suppression of carbon fixation should therefore be viewed as a protective mechanism that maintains the long‑term capacity of bryophyte communities to fix carbon and sustain biogeochemical cycling under stress.

The synergistic variation in nutrient traits of epilithic bryophytes was characterized by increases in N, P, and Ca and decreases in C:N, C:P, and N:P (**Figures 4**, **5**). This pattern contradicts the predictions of the classic growth rate hypothesis under nutrient-sufficient conditions (Niklas, 2006). In rocky habitats of degraded karst regions, soil nutrients are extremely deficient and represent the primary limiting factor constraining ecosystem productivity (Zhang et al., 2015). As important participants in nutrient cycling (Brown and Bates, 1990), bryophytes mainly obtain their elements from atmospheric deposition and growth substrates (e.g., soil, rock), and their elemental contents are affected by regional spatial conditions and ecosystems (Eckstein, 2000). Previous studies have demonstrated that bryophytes possess a high nitrogen recovery efficiency; in nutrient-poor environments, they can reabsorb and utilize 50–60% of the nitrogen from decaying organic matter, thereby effectively retaining nutrients in barren environments with limited soil contact that rely on atmospheric deposition (Aldous, 2002; Bates, 1994). On this basis, we infer that in nutrient-poor, degraded karst ecosystem, epilithic bryophytes can efficiently resorb N and P from decaying organic matter. They can also capture and retain nutrient elements from atmospheric deposition via their dense prostrate growth form and promote internal nutrient cycling within the community (Bates, 1992), thereby facilitating the accumulation of nutrients. In addition, bryophytes may prioritize the allocation of their limited carbon resources to systems for nutrient acquisition and retention rather than to growth. Furthermore, bryophytes lack a long-distance carbon transport system, and the accumulation of photosynthetic products within cells will further influence the variation in the C:P ratio (Wang et al., 2015). Notably, N was significantly positively correlated with P (*P* < 0.05, **Figure 2**). The interaction between N and P can promote phosphorus uptake by plants (Schleuss et al., 2020), and under N-limiting conditions, plants may upregulate P uptake pathways in response to N availability (Zeng et al., 2012), thereby optimizing resource acquisition in stressful environments.

This study further supports the previous view that the response of bryophytes to environmental signals is essentially an adjustment to their energy status (Mohanasundaram and Pandey, 2022). Among these, C:P and C:N ratios serve as the key regulatory signals for bryophytes to coordinate growth, maintenance, and defense strategies in fluctuating environments. The synergistic variation pattern of traits observed in this study indicates that changes in C:P and C: N ratios reflect the allocation trade-off of carbon resources between nutrient acquisition and retention systems and physical defense structures, thereby driving plastic responses of bryophytes in morphology, developmental stage, and physiology. This mechanism is of key evolutionary significance for bryophytes to adapt to diverse habitats (Thelander et al., 2005; Sugiyama et al., 2012). These discussions and analyses provide strong support for our second hypothesis. Furthermore, the ecological functional changes induced by the adjustment of ecological strategies in epilithic bryophytes may bring positive effects and certain ecological benefits to degraded karst ecosystems (Tu et al., 2021). Previous studies have demonstrated that bryophytes retain most nutrients within their own systems via high resorption efficiency and exhibit a slow decomposition rate, thereby acting primarily as nutrient reservoirs in ecosystems (Eckstein, 2000; Liu et al., 2020). Based on our observations of trait patterns indicative of potential nutrient immobilization under stress conditions, we hypothesize that this adaptive strategy at the individual level may exert cascading effects at the community and ecosystem scales. Specifically, if these trait shifts indeed translate into higher nutrient resorption efficiency and slower decomposition, they could influence nutrient retention and cycling in degraded ecosystems. However, we emphasize that this remains a hypothesis derived from trait-based inference. In future work, we plan to conduct long-term monitoring and controlled experiments to test these predictions.

## 5 Conclusions

As pioneer group in degraded karst ecosystems, epilithic bryophytes are crucial in understanding the impacts of environmental changes on plant communities and ecosystem functions through their variation patterns of functional traits and adaptive mechanisms. Our study demonstrates that intraspecific trait variation of epilithic bryophytes in the study area, like interspecific variation, can significantly shape community functional composition, revealing a pattern of “physiological convergence – morphological differentiation”. Temperature, canopy density, and light intensity were the key environmental factors associated with trait variation. Under combined stress, nutrient-related traits of epilithic bryophytes (C:P, C:N, P) acted as hub traits linking environmental stress, individual physiology, and ecosystem nutrient cycling. Epilithic bryophytes formed a conservative adaptive strategy to stressful environments in degraded karst ecosystem via trade‑offs and combinations between morphological traits and photosynthetic physiological traits. We infer that this synergistic variation pattern of epilithic bryophytes may enhance their soil nutrient retention capacity and could contribute positively to the ecological restoration of degraded karst ecosystems, although direct experimental validation is required to confirm these effects. These ecological benefits are anticipated to make substantial potential contributions to vegetation restoration and sustainable development in karst regions.

## Data Availability

The raw data supporting the conclusions of this article will be made available by the authors, without undue reservation.
