# Supplementary material for: Functional traits variation and adaptive strategies of epilithic bryophytes in degraded karst ecosystem
Source: Front Plant Sci. 2026 Sep 7;17:1847125. doi: 10.3389/fpls.2026.1847125 (PMC13590753; doi:10.3389/fpls.2026.1847125)
Supplement: Supplementary file 1 [file DataSheet1.pdf]

Supplementary Material

Table S1. Geographic coordinates and environmental characteristics of the 25 sampling plots in Nidang Stone Forest, Guizhou, China.

Degree of rocky desertification	Longitude and latitude of site center	Elevation /m	Rock exposure rate /%	Aspect	Slope /°	Slope position of sampling point	Dominant vascular plants
None rocky Desertification	104°49′22.43″E, 24°48′11.32″N	1184	15	North-facing slope	22	Lower	*Eucalyptus robusta*
	104°49′13.46″E, 24°48′02.18″N	1249	12	North-facing slope	29	middle	*Larix gmelinii*、*Casuarina equisetifolia*、*Platycladus orientalis*、*Miscanthus sinensis*
	104°49′11.57″E, 24°47′56.97″N	1266	10	North-facing slope	23	upper	*Eleutherococcus trifoliatus*、*Eupatorium coelestinum*、*Nerium oleander*
	104°49′47.86″E, 24°48′25.38″N	1183	8	North-facing slope	35	middle	*Broussonetia papyrifera*
	104°50′18.80″E, 24°48′33.98″N	1135	11	South-facing slope	30	middle	*Alocasia odora*
Potential Rocky Desertification	104°49′15.33″E, 24°48′05.02″N	1244	22	North-facing slope	22	lower	*Asplenium trichomanes*、*Mallotus barbatus*
	104°49′14.88″E, 24°48′03.96″N	1257	24	South-facing slope	33	middle	*Crassocephalum crepidioides*、*Pothos chinensis*
	104°49′08.76″E, 24°48′05.16″N	1291	25	North-facing slope	30	upper	*Lonicera japonica*、*Broussonetia monoica*
	104°49′59.34″E, 24°48′17.03″N	1130	22	South- facing slope	33	lower	*Ficus religiosa*、*Rhus chinensis*
	104°50′17.24″E, 24°48′23.14″N	1138	28	North-facing slope	28	middle	*Eriobotrya japonica*、*Cunninghamia lanceolata*、*Cinnamomum camphora*
Light Rocky Desertification	104°49′19.46″E, 24°48′10.41″N	1202	36	South-facing slope	35	lower	*Pleuropterus multiflorus*、*Dicranopteris pedata*、*Paederia cruddasiana*
	104°49′18.14″E, 24°48′08.82″N	1205	40	North-facing slope	56	lower	*Phytolacca acinosa*、*Carpesium abrotanoides*
	104°49′10.25″E, 24°48′01.65″N	1253	35	South-facing slope	5	upper	*Larix gmelinii*、*Platycladus orientalis*
	104°51′27.28″E, 24°49′37.68″N	1272	38	South-facing slope	32	middle	*Rubus peltatus*、*Amorphophallus konjac*

	104°50′53.62″E, 24°49′06.17″N	1105	45	South-facing slope	30	middle	*Callicarpa bodinieri*、*Rosa multiflora*
Moderate Rocky Desertification	104°49′15.28″E, 24°48′07.67″N	1219	65	South-facing slope	44	lower	*Ulmus pumila*、*Erigeron canadensis*
	104°49′07.75″E, 24°47′56.97″N	1266	68	North-facing slope	23	upper	*Eleutherococcus trifoliatus*、*Nerium oleander*
	104°49′05.05″E, 24°48′00.18″N	1278	55	North-facing slope	27	upper	*Sinomenium acutum*
	104°51′09.40″E, 24°49′07.31″N	1114	65	North-facing slope	44	middle	*Osmanthus fragrans*
	104°50′20.50″E, 24°48′56.98″N	1111	60	South-facing slope	46	lower	*Alangium chinense*、*Boehmeria silvestrii*、*Pteridium aquilinum*
Severe Rocky Desertification	104°49′09.96″E, 24°48′03.05″N	1259	90	South-facing slope	34	middle	*Amaranthus tricolor*
	104°49′04.15″E, 24°48′02.60″N	1280	85	North-facing slope	26	upper	*Ageratina adenophora*、*Sinomenium acutum*、*Erigeron canadensis*
	104°49′07.63″E, 24°48′02.02″N	1273	92	North-facing slope	31	upper	*Abutilon theophrasti*、*Clerodendrum bungei*
	104°51′18.73″E, 24°49′38.67″N	1301	95	South-facing slope	41	upper	*Platycarya strobilacea*、*Hibiscus mutabilis*、*Mirabilis jalapa*
	104°49′48.50″E, 24°48′32.39″N	1134	90	South-facing slope	40	lower	、*Erigeron canadensis*

Table S2. Species composition and important values of epilithic bryophytes in different habitats in Nidang Stone forest.

Family	Genus	Species	Importance value				
			NRD	PRD	LRD	MRD	SRD
Thuidiaceae	*Cyrto-hypnum*	*Cyrto-hypnum gratum*			0.017		
	Claopodium	*Claopodium gracillimum*		0.033	0.017		
	Thuidium	*Thuidium assimile*			0.017		
Pottiaceae	*Bryoerythrophyllum*	*Bryoerythrophyllum alpigenum*	0.033				
	Weissia	*Weissia controversa*	0.067				

Hypnaceae	*Eurohypnum*	*Eurohypnum leptothallum*	0.067		0.033	0.016	0.017
	Isopterygium	*Isopterygium serrulatum*				0.016	
	Gollania	*Gollania japonica*			0.017	0.017	0.017
Entodontaceae	*Entodon*	*Entodon luridus*		0.033			
		E. obtusatus				0.017	
		E. cladorrhizans			0.016		
		E. longifolius	0.033				
Meteoriaceae	*Meteoriopsis*	*Meteoriopsis squarrosa*		0.016			
	Meteorium	*Meteorium miquelianum*	0.200	0.167	0.100	0.167	0.233
		M. subpolytrichum	0.017	0.067	0.033	0.100	0.017
		M. papillarioides	0.016	0.067	0.017	0.033	0.033
		M. cucullatum		0.016			0.017
Ditrichaceae	*Trichostomum*	*Trichostomum brachydontium*		0.100	0.033	0.017	
Anomodontaceae	*Anomodon*	*Anomodon viticulosus*	0.017		0.017	0.067	
		A. minor	0.100	0.100	0.167	0.200	0.267
		A. rugelii	0.016	0.017	0.067	0.016	
Neckeraceae	*Neckera*	*Neckera pennata*				0.067	0.016
Brachytheciaceae	*Brachythecium*	*Brachythecium plumosum*		0.017			0.033
		B. pulchellum		0.017			
		B. dicranoides	0.033	0.017	0.017	0.017	
		B. moriense	0.034	0.017			
		Brachythecium reflexum			0.016		
		B. perminusculum			0.016		0.033

		B. populeum	0.034				
		B. garovaglioides					0.033
		B. helminthocladum		0.034			
	Homalothecium	*Homalothecium leucodonticaule*	0.133	0.200	0.200	0.100	0.133
	Palamocladium	*Palamocladium nilgheriense*	0.133	0.016	0.100	0.033	0.067
		P. euchloron		0.033	0.066	0.033	
	Myuroclada	*Myuroclada maximowiczii*					0.033
	Eurhynchium	*Eurhynchium striatulum*				0.033	
Ptychomitriaceae	*Ptychomitrium*	*Ptychomitrium polyphylloides*					0.017
Bryaceae	*Bryum*	*Bryum caespiticium*	0.034				
Pseudoleskeellaceae	*Pseudoleskeella*	*Pseudoleskeella catenulata*			0.017	0.017	0.017
Mniaceae	*Plagiomnium*	*Plagiomnium acutum*			0.017		
Leskeaceae	*Pseudoleskeopsis*	*Pseudoleskeopsis zippelii*				0.017	
Dicranellaceae	*Dicranella*	*Dicranella micro-divariata*				0.017	0.017
Sematophyllaceae	*Gammiella*	*Gammiella ceylonensis*		0.033			
Racopilaceae	*Racopilum*	*Racopilum cuspidigerum*	0.033				

Table S3. Bootstrap robustness of network topology.

Metric	Original value	Bootstrap mean (n=1000)	SD
Edge Density	0.0968	0.0960	0.0044
Modularity (Q)	0.736	0.7333	0.0289

Table S4. Occurrence frequency of each edge across 1000 bootstrap resamples.

Trait-A	Trait-B	Bootstrap-Occurrence	Trait-A	Trait-B	Bootstrap-Occurrence
SH	BCW	1	Mg	C:P	1
LL	LW	1	C:N	C:P	1
LL	LA	1	C:P	N:P	1
LW	LA	1	SH	C	0.998
SER	SCR	1	MC L/W	BCL	0.995
MCW	MCL	1	Mg	N:P	0.995
MCW	MC L/W	1	C	K	0.994
MCL	MC L/W	1	K	N:P	0.994
BCL	BC L/W	1	SH	C:N	0.982
BCW	BC L/W	1	MC_HWT	MCL	0.981
Fv/Fm	ΦPSII	1	C	C:P	0.98
C	Mg	1	MCL	BCW	0.973
C	C:N	1	K	C:N	0.972
N	P	1	P	Mg	0.97
N	Ca	1	LW	L L/W	0.888
N	C:N	1	qP	ΦPSII	0.782
N	C:P	1	MC_HWT	MC L/W	0.77
P	C:N	1	MCL	BCL	0.769
P	C:P	1	BC_HWT	BC L/W	0.619
P	N:P	1	BC_HWT	BCL	0.617
K	Mg	1	SH	Mg	0.596

K	C:P	1	MC L/W	BCW	0.496
Mg	C:N	1			

Table S5. β diversity index of epilithic bryophytes in Nidang Stone forest different rocky desertification habitats.

method	habitats	PRD	LRD	MRD	SRD
C_s	NRD	0.53	0.36	0.36	0.28
	PRD		0.44	0.39	0.32
	LRD			0.52	0.36
	MRD				0.51
β_c	NRD	9.00	12.50	12.50	13.00
	PRD		11.50	12.50	13.00
	LRD			10.00	12.50
	MRD				9.50

Table S6. Variance explained by principal components.

Component	Eigenvalue	Variance explained (%)	Cumulative (%)
PC1	3.52	50.24	50.24
PC2	1.28	18.30	68.54
PC3	1.01	14.42	82.96

PC4	0.79	11.27	94.23
PC5	0.20	2.91	97.14
PC6	0.15	2.14	99.28
PC7	0.05	0.72	100.00

Table S7. Loadings of environmental variables on the first principal components.

Variable	PC1
Elevation	0.076
TRASP	0.111
CD	-0.496
Ta	0.378
Ha	-0.377
RSTa	0.488
Light	0.461
